# 3D Pharmacophore-Based Virtual Screening and Docking Approaches toward the Discovery of Novel HPPD Inhibitors

**DOI:** 10.3390/molecules22060959

**Published:** 2017-06-09

**Authors:** Ying Fu, Yi-Na Sun, Ke-Han Yi, Ming-Qiang Li, Hai-Feng Cao, Jia-Zhong Li, Fei Ye

**Affiliations:** 1 Department of Applied Chemistry, College of Science, Northeast Agricultural University, Harbin 150030, China; fuying@neau.edu.cn (Y.F.); sunyina9@163.com (Y.-N.S.); yikehan@neau.edu.cn (K.-H.Y.); mqli@neau.edu.cn (M.-Q.L.); caohf@neau.edu.cn (H.-F.C.); 2 School of Pharmacy, Lanzhou University, 199 West Donggang Rd., Lanzhou 730000, China; lijiazhong@lzu.edu.cn

**Keywords:** HPPD inhibitors, pharmacophore model, molecule docking, HipHop model, virtual screening, ChemDiv

## Abstract

*p*-Hydroxyphenylpyruvate dioxygenase (HPPD) is not only the useful molecular target in treating life-threatening tyrosinemia type I, but also an important target for chemical herbicides. A combined in silico structure-based pharmacophore and molecular docking-based virtual screening were performed to identify novel potential HPPD inhibitors. The complex-based pharmacophore model (CBP) with 0.721 of ROC used for screening compounds showed remarkable ability to retrieve known active ligands from among decoy molecules. The ChemDiv database was screened using CBP-Hypo2 as a 3D query, and the best-fit hits subjected to molecular docking with two methods of LibDock and CDOCKER in Accelrys Discovery Studio 2.5 (DS 2.5) to discern interactions with key residues at the active site of HPPD. Four compounds with top rankings in the HipHop model and well-known binding model were finally chosen as lead compounds with potential inhibitory effects on the active site of target. The results provided powerful insight into the development of novel HPPD inhibitors herbicides using computational techniques.

## 1. Introduction

Weeds compete with crops for sunshine, water, nutrients, and space, which influences the growth of crops and undermines both crop quality and yield. In agrochemical research, the discovery of novel high-activity and low-toxicity herbicide lead compounds still remains a challenge. 4-Hydroxyphenylpyruvate dioxygenase belongs to the non-heme Fe(II)-dependent dioxygenase family [1,2]. As an important enzyme correlated to the pigment synthesis and tyrosine catabolism in most organisms, HPPD is important in drug discovery in both agricultural and therapeutic areas [3,4,5]. HPPD catalyzes the conversion of 4-hydroxyphenylpyruvate (HPPA) to homogentisate (HGA), and this transformation involving decarboxylation, aromatic hydroxylation, and substituent migration in a single catalytic cycle is unique in Nature [6]. In plants, HGA can be further transformed into tocopherol and plastoquinone, both of them are crucial for the normal growth of plants [7]. Inhibition of HPPD will block photosynthesis, which leads to the deficiency in isoprenoid redox cofactors such as plastoquinone and tocopherol, and finally causes growth inhibition, necrosis and death of treated plants [8,9,10,11].

Herbicides which inhibit HPPD, represent one of the newest classes of agrochemicals available for use in crop production [12]. HPPD-inhibiting herbicides show many advantages, such as low application rates, low toxicity, broad-spectrum weed control (including herbicide-resistant weed biotypes), excellent crop selectivity and benign environmental effects [13,14]. Several of them are currently used as selective broad leaf herbicides including triketones, pyrazoles, isoxazoles, diketone nitriles and benzophenones [15,16]. The triketone herbicides have spurred a variety of commercialized HPPD inhibitors through chemical modification, such as sulcotrione, mesotrione and benzobicylon [17,18,19], but the main problem associated with the use of herbicides is the occurrence of herbicide-resistant weeds. Therefore, it is necessary to develop efficient herbicides with novel structures actives against HPPD.

The use of computational techniques in drug discovery and development has become the most effective method. Among them, virtual screening is a conventional method used in drug discovery, which screen large collections of compounds to identify molecular structures that are most likely to bind into a particular biological target [20]. It has been reported that molecular docking, pharmacophore modeling, and structure-based virtual screening have been successful applied in drug discovery. Structure-based virtual screening has emerged as an efficient strategy in identifying potential a natural product-like STAT3 dimerization inhibitor from a database of natural product and natural product-like compounds, and molecular docking analysis suggested that compound **1** if the structure is not shown calling the compound “1” is useless—give name of structure in a figure to identify might putatively function as an inhibitor of STAT3 dimerization by binding to the SH2 domain [21]. Novel TLR1–TLR2 inhibitors were obtained through molecular docking from a database of natural product and natural product-like compounds and the results of activity experiments show that compound **1** was the most effective in inhibiting TNF-a and IL-6 secretion induced by Pam3CSK4 in RAW 264.7 cells [22]. High-throughput, ligand-docking based virtual screening methods were applied to identify small agents targeting menin–MLL binding from a natural product/natural product-like chemical database. From the activity assay, compound **1** which was tested in a bimolecular fluorescence complementation (BiFC) assay emerged as the top candidate for inhibiting menin–MLL interaction. Moreover, a high degree of shape complementarity is observed between compound **1** and the binding pocket of menin, suggesting that this protein–ligand interaction could also be stabilized by significant hydrophobic interactions [23]. Rutin, as a promising lead compound, would be further developed into an antidyslipidemic molecule as a good alternative to statins using a docking-based strategy and MD stimulation [24]. A metadynamics-based protocol was developed to investigate the unbinding mechanism of an inhibitor of the pharmacologically relevant target p38 MAP kinase. The calculation results showed that the salvation of the ligand and of the active site played crucial roles in the unbinding process and demonstrating that metadynamics could be a powerful tool in designing new drugs with engineered binding/unbinding kinetics [25].

**Figure 1:**
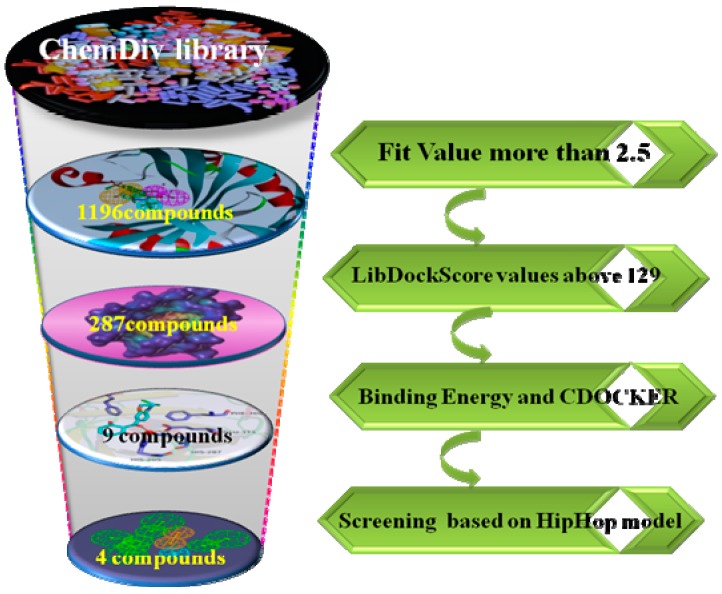
The workflow of virtual screening.

The virtual filtered strategy graph is shown in Figure 1. The goal of this study is to identify the novel and potential structure of HPPD inhibition through 3D pharmacophore models based on the known crystal complex of HPPD (PDB ID: 1TFZ). CBP-Hypo2 with quality = 0.721 (Fair) was selected as the best hypothesis, which included one hydrogen bond donor (HBD), one ring aromatic (RA) and two hydrophobic features (HY). Subsequently, the reliable pharmacophore hypotheses were used in virtual screening ChemDiv databases to identify potential HPPD inhibitors. The virtual screened hit compounds were then docked into active pocket of HPPD in DS2.5. Further, the selected screened hits were performed binding energy calculation and precision docking. Nine compounds with good affinity were obtained. The nine hits obtained were matched to HipHop model. Finally, four compounds displayed good match to ligand-based pharmacophore HipHop-hypo2.

## 2 Results and Discussion

### 2.1. Pharmacophore Model Generation and Validation

**Figure 2:**
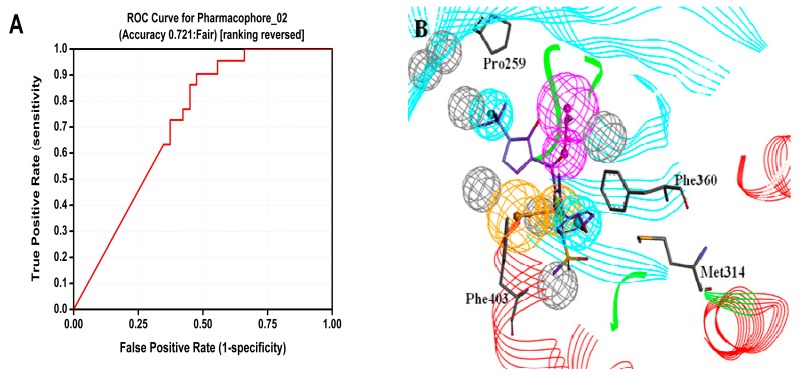
Generation of CBP model for HPPD inhibitors (**A**) ROC curve of CBP-Hypo2; (**B**) Complex based pharmacophore (CBP) model with co-crystallized ligand, magenta, orange and cyan represents HBD, RA and HY respectively.

CBP that was generated based on protein 1TFZ and inhibitor DSA869 was used as a virtual screening model to discovery novel HPPD inhibitors. Twenty two active compounds and 38 inactive compounds were used as testing set to validate the receptor-ligand pharmacophore automatically. Six hypotheses were generated. As shown in Figure 2, CBP-Hypo2 desired Quality (0.712, Fair) was selected as the best hypothesis. One hydrogen bond donor (HBD), one ring aromatic (RA) and two hydrophobic features (HY) were regarded as the critical features of the model. During HipHop pharmacophore generation, six highly active inhibitors were selected from the literature to serve as training set (Table 2). Ten hypotheses were generated and ordered by ranking score. All 10 hypotheses ranked scores ranging from 81.54 to 72.16 (Table 3). The rank values and feature pharmacophore of HipHop-Hypo1 were same as HipHop-Hypo2.

**Figure 3:**
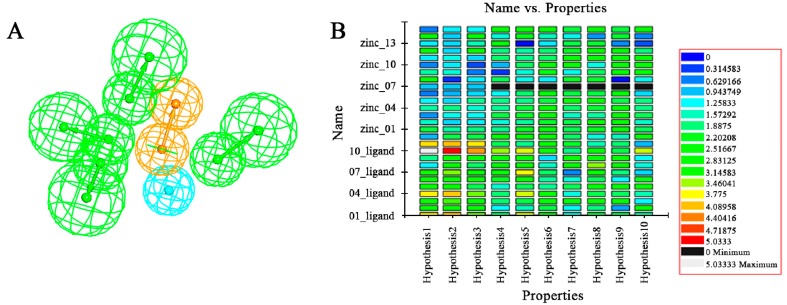
Generation of the HipHop pharmacophore. (**A**) The HipHop-Hypo2 chemical features. The color of the pharmacophore features, namely, HBA, RA and HY, re green, orange and cyan, respectively; (**B**) The heat map of the ten hypotheses from the test.

The Receiver Operating Characteristic (ROC) curve was used to evaluate the degree of false positivity of the model screening compound. The curve was obtained by plotting false positive rate for x-axis against true positive rate on y-axis in Figure 2. The accuracy of the test was shown by measuring the area under the curve (AUC). The result of the model represented with excellent AUC score of 0.721. For the HipHop model, a test composed of active compounds and inactive compounds was used evaluate the selective model. HipHop-Hypo2 was considered as the best chemical hypothesis due to the fact the model was better at distinguishing active and inactive compounds. As can be seen from the Figure 3, the Fit values of the 12 active compounds were above 2.0, compared to the inactive molecules distributed in blue area.

### 2.2. CBP Pharmacophore Model-Based Virtual Screening

**Figure 4:**
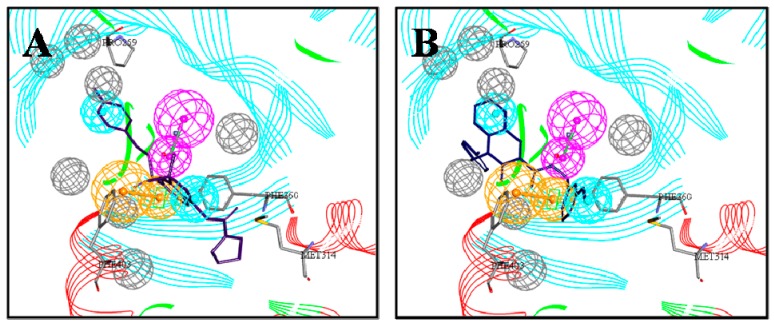

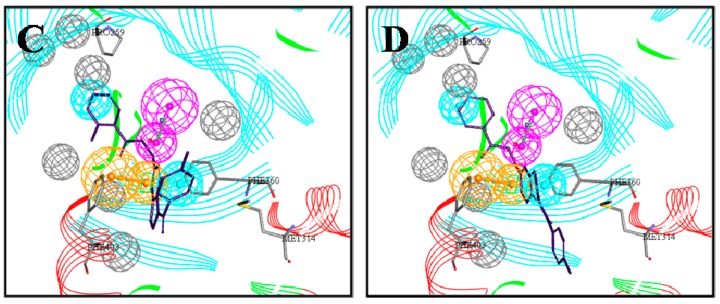
Mapping of each of the best hits to CBP-Hypo2. The color of the pharmacophore features, namely, HBD, RA and HY are pink, orange and cyan, respectively. (**A**) Compound L503-0533; (**B**) compound G622-0791; (**C**) compound G883-0326 (**D**) compound G883-0470.

Initially, the CBP model was used as a query to search the ChemDiv databases with 151047 compounds. Fit Value is a measure of the overlap between the features in the pharmacophore and chemical features in the molecule, which helps understand the chemical meaning of the pharmacophore hypothesis [26]. According to the Fit Value greater than 2.5, 1196 hit compounds mapping onto the pharmacophore model CBP-Hypo2 were retrieved, which included some compounds structurally similar to existing HPPD inhibitors and some novel scaffolds. As shown in Figure 4, the obtained compounds were well matched to the CBP model and formed *π*-*π* interactiona with the Phe360 and Phe403 residues. Simultaneously, residues Pro259 and Met314 generated hydrophobic interactions with the aromatic ring or methyl.

### 2.3. Molecular Docking

**Figure 5:**
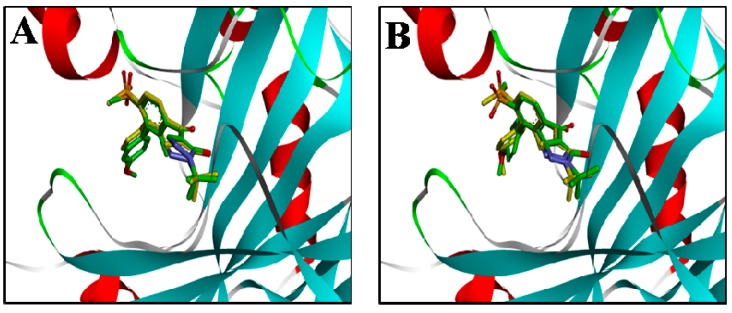
Alignment of the docked ligands with the ligands in the crystallographic complex. (**A**) The ligand by the LibDock docking method; (**B**) the ligand by the CDOCKER docking method. Docked ligands are green, the ligand in the crystallographic complex is yellow.

In order to reduce the number of false positive screened virtual hits, docking analysis was performed at the active site of *At*HPPD using DS2.5. The ligand in the protein 1TFZ was extracted and hydrogen atoms were added. The docking method was carried out applying two docking methods, which were LibDock and CDOCKER. Subsequently, binding poses of the docking compound were compared with the ligand in the crystallographic complex and RMSD values of 0.74 and 0.55 were calculated, respectively. As can be seen from Figure 5, the ligands docked by the two docking methods could be well aligned with the ligands in the crystallographic complex, so the two methods demonstrated the accuracy and reliability of the docking.

**Figure 6:**
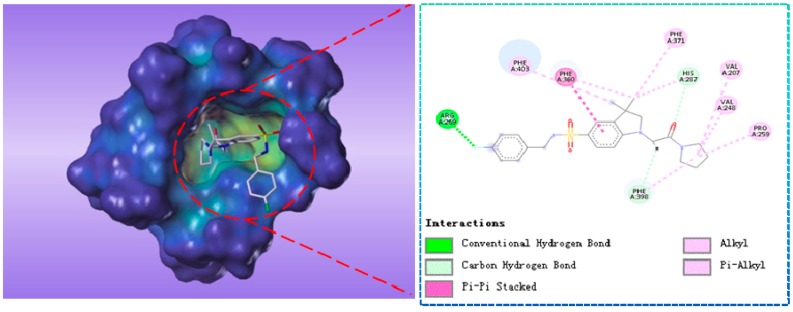
The receptor-ligand interaction of screening compound L503-0533 with the HPPD active site.

These virtual 1196 molecules retrieved after pharmacophore-based screenings were subjected to receptor-based virtual screening by using LibDock methods. Docking experiments was applied to compare the binding affinities of known inhibitors with that of the screened hits and to rank the screened hits on the basis of interactions with amino acid residues of the active site. 287 Hit compounds were chosen that showed LibDockScore values above 129. Further, the selected screened hits were subsequently submitted for their binding energy value calculation and precision docking in the ‘Calculate Binding Energies module’ and ‘CDOCKER module’ of DS2.5, respectively. Finally, according to binding modes, binding affinity, nine hit compounds with the highest docking score and lowest binding energy were selected as the target hits. The different significant chemical interactions, viz., π-alkyl, *π*-*π*, hydrogen bonds, etc., of the best hits have been presented in the following figures. As shown in Figure 6, for compound L503-0533, Phe360 generated *π*-*π* interactions with the benzene ring and Arg269 interacted with the fluorine via a hydrogen bond.

**Figure 7:**
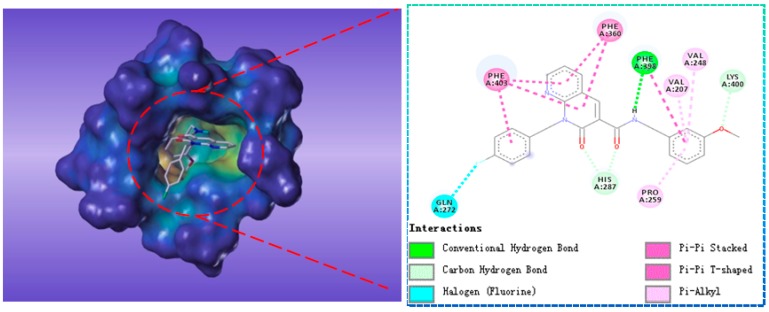
The receptor-ligand interaction of screening compound G622-0791 with the HPPD active site.

Compound G622-0791 was found to fully embed into the active pocket (Figure 7), and interacted with amino acids Gln272, Phe398 and Lys400 via H-bonds, meanwhile, the two benzene rings formed two pairs of sandwiches interacting with Phe360 and Phe403 at the binding site.

**Figure 8:**
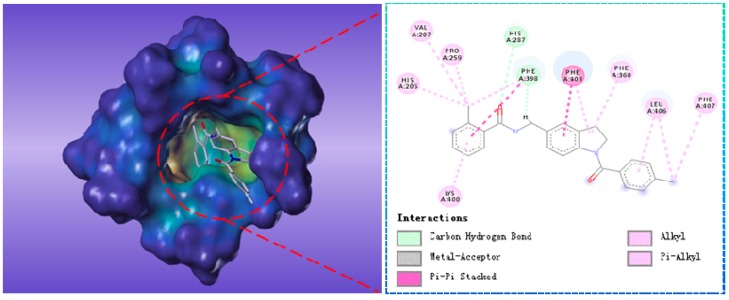
The receptor-ligand interaction of screening compound G883-0326 with the HPPD active site.

**Figure 9:**
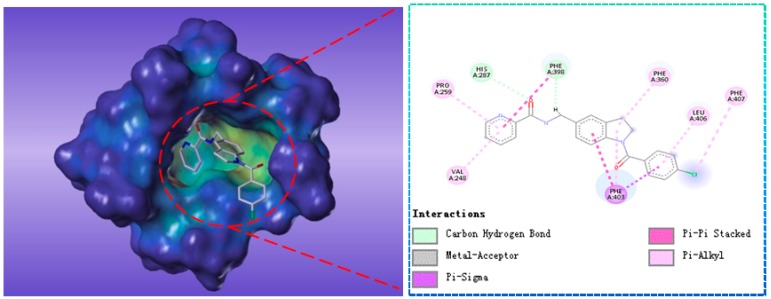
The receptor-ligand interaction of screening compound G883-0470 with the HPPD active site.

Compound G883-0470 formed *π*-*π* stacking interactions with Phe398, Phe403 and Phe406 and generated hydrogen bond interactions with His287 and Phe398 as depicted in Figure 8. Compound G883-0326 formed *π*-*π* stacking with benzyl ring of Phe398, Phe403 and Phe360. His287 interacted with carbonyl via hydrogen bond was shown in Figure 9.

### 2.4. HipHop Pharmacophore Model-Based Virtual Screening

**Figure 10:**
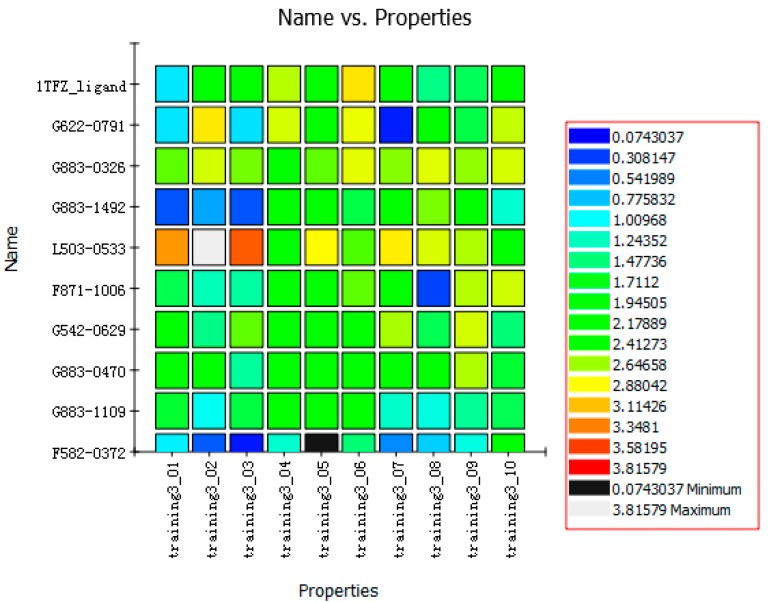
Heat map of the ten hypotheses from docked compounds and ligand of HPPD.

The nine compounds obtained were matched to the HipHop model in the Figure 10, two figures with same number and the results indicated that four compounds were well matched to the ligand-based pharmacophore HipHop-Hypo2 and all the colors of the other five compounds with low fit values in the heat map were light blue. Compound L503-0533 exhibited the highest matching value of 3.8. Finally, four new compounds with diverse scaffolds were selected as possible candidates for the designing of potent HPPD inhibitors (Table 1). The values of the four compounds were higher than those of the reference compound with Binging Energy, LibDockScore -CDOCKER ENERGY, Fit Value. The compound G622-0791 was finally selected as the most potent HPPD inhibitor based on its least binding energy (−167.41 kcal/mol). The -CDOCKER score of this compound was −39.18 with a Fit Value (pharmacophore-based on CBP-Hypo2) of 2.97.Further investigations on these four compounds involving testing in vitro and in vivo against HPPD are currently underway in our laboratories.

## 3. Materials and Methods

### 3.1. Data Collection and Preparation

The X-ray crystal structure of complex HPPD with an inhibitor (PDB code: 1TFZ) was downloaded from the RCSB Protein Data Bank (www.rcsb.org). The inhibitor was removed from the complex as an active ligand in building the CBP models.

Virtual screening using the ChemDiv database was performed to identify novel potential HPPD inhibitors. Based on the published literatures [27,28,29], 22 HPPD inhibitors with IC_50_ less than 0.1 nM were collected as the active ligands of CBP for verifying the model. Meanwhile, to validate the selectivity of the obtained pharmacophore models, 38 compounds randomly selected from the HPPD-decoys set were chosen as the inactive ligands.

Based on the known HPPD inhibitor [29,30,31] six typical compounds, as the training set in the HipHop model, were used to generate common feature based pharmacophore models. The bioactivity value IC50 and molecular properties of these compounds were listed in Table 2. Twenty seven compounds were selected as a test set, among which 12 compounds were HPPD inhibitors with .ligand file, and 17 compounds randomly obtained from the ZINC database were used as inactive molecules with .zinc files. All the molecules were prepared and optimized using SYBYL-X 2.0.

### 3.2. Pharmacophore Model Generation

The CBP model was developed within CBP module in Catalyst using the known crystal complex of HPPD (PDB ID: 1TFZ) and ligand (DSA869), and both were chosen as the receptor and ligand, respectively. Validation option was set as True. 22 Reported active HPPD inhibitors and 38 inactive compounds were used as the active ligands and inactive ligands, respectively. The remaining parameters were set to default values. Six hypotheses were generated, and CBP-Hypo2 with desired Quality (0.721, Fair) was subsequently used to screen the library.

HipHop pharmacophore models were generated from a set of known molecules with promising activity towards HPPD. Based on the atom-types presented in the molecule, HipHop selected the key common chemical features for creating 3D-pharmacophore models. The principal value 1 for all the ligands and maximum-omit feature as 2. The common features pharmacophore generation module “*Feature Mapping*” was used to identify the important chemical features of the training set compounds before building HipHop pharmacophore model. Hydrogen bond acceptor (HBA), hydrogen bond donor (HBD), hydrophobic features (HY) and ring aromatic (AR) were considered for generation of the pharmacophore model. The diverse conformation option was applied and 250 conformations within 20 kcal·mol^−1^ cutoff were generated using the “BEST”. The final common feature 3D-pharmacophore models were ranked based on pharmacophore fit value. The fit value of the ten chemical hypothesis generated along with the key 3D-pharmacophoric chemical features are presented in Table 3.

Validating the hypothesis is one of the significant methods in pharmacophore generation. Test sets including active compounds and inactive compounds was prepared using the same protocol as the training set preparation and used to determine whether the hypothesis was able to discern active compounds. Fit value was used as an important evaluation criterion [32].

### 3.3. Pharmacophore-Based Virtual Screening

About 151,047 small molecules were obtained from the ChemDiv database (www.chemdiv.com) and subjected to virtual screening. All the compounds which were optimized in DS2.5 were used as virtual screening library. Subsequently, the fist screening of CBP-Hypo2 was used to retrieve the database in ‘Search, Screen and Profile module’ of DS2.5. The number of conformations was set to 200, while the conformation method was set to BEST, which provided a complete and improved coverage of the conformational space by performing a rigorous energy minimization and optimizing the conformations in both torsional and Cartesian space by the poling algorithm [33]. Minimum Interfeature Distance was set to 2. Limit Hits was set to First N, and Maximum Hits was set to 500. The rest parameters were set to default values.

### 3.4. Molecular Docking

The *At*HPPD crystal structure (PDB ID: 1TFZ) with resolution of 1.8 Å was used for molecular docking studies. The protein was prepared by removing the water and some other co-crystallized small molecules, and potentials were assigned using CHARMm force field, the missing atoms residues were building using the ‘Build and Edit Protein’ module, and cleaning protein were prepared in ‘Prepare Protein module’. The protein structure was energy minimized for 5000 steps (with the heavy atoms constrained) using the conjugate gradient algorithm with the ‘Minimize and Refine Protein’ module in DS2.5. After the protein preparation, the binding site of the protein was defined based on volume occupied by the known ligand pose already in an active site. The obtained receptor was used as the “Input Receptor Molecule” parameter. All hit compounds subjected to fist filtering processes were saved as .sd file. The saved structures were chosen as “Input Ligand” and docked into the active site of HPPD. Docking was performed to ensure the proper binding orientation and placement of each ligand and to confirm the geometric fit of each ligand inside the active site. During the docking process top 10 conformations were saved for each ligand based on dock score value after the energy minimization using the smart minimize method through LibDock and CDOCKER methods.

## 4. Conclusions

In this study, a strategy for the selection of new chemical compounds with HPPD inhibition by virtual screening was performed. Virtual screening was divided into receptor-based virtual screening and ligand-based virtual screening. Receptor-based virtual screening was more effective in detecting novel chemical scaffolds and is more commonly used in academic labs.

The CBP model was generated based on the HPPD enzyme receptor and active ligands that extracts the essential structural features required for inhibition, which was helpful for screening of novel molecules having inhibitory activity against HPPD based on receptor, CBP-Hypo2 was used to screen the ChemDiv library to find potential HPPD inhibitors and Fit Value was selected as an important criterion. The hydrophobic groups (benzene and methyl) of the four compounds obtained from the ChemDiv database formed hydrophobic interactions with Met314 and Pro205, and the intermediate aromatic ring generated *π*-*π* interactions with Phe403 and Phe360. Further, molecular docking was performed to provide insights into molecular recognition via protein–ligand interactions. The result was analyzed based on the docking score, binding modes, and molecular interactions with active site residues. Subsequently, the binding free energy of selected compounds relevant to ligand and receptor was calculated, and nine novel scaffold hits with good docking scores and low binding energy were chosen. The screened compounds could be completely embedded into the HPPD active pocket and interact with the Phe360, Phe403, Arg269, Phe398 and Asn402 residues of the active site and so on. Finally, compounds obtained through docking were matched with a HipHop model, and four hits with high Fit value were identified that could be used as potential leads for further optimization in designing new HPPD inhibitor herbicides. This study provided a set of guidelines that will greatly help in designing novel and more potent HPPD inhibitors herbicides.

## Figures and Tables

**Table 1:** The 2D structure of the obtained compound and the evaluation value.

Name	Structure	Binging Energy	LibDock Score	-CDOCKER Energy	Fit Value
Crystallographic ligand	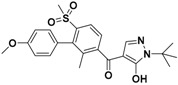	−68.857	130.542	21.08	2.35
L503-0533	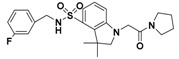	−130.39	151.48	31.75	2.75
G622-0791	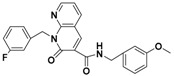	−167.41	138.71	39.18	2.97
G883-0326	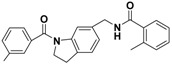	−125.71	141.43	21.93	2.56
G883-0326	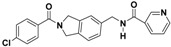	−133.97	138.34	22.73	3.02

**Table 2:** Chemical structures and molecular properties of the training set compounds in HipHop model and screening compounds.

Structure	AlogP	Weight	Num-H Acceptors	Num-H Donors	Num-H Rotatable Bonds	Molecular Polar Surface Area	IC_50_
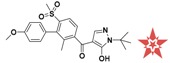	3.429	444.54	7	2	6	104.32	-
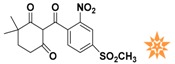	2.53	329.23	5	0	4	97.03	0.28
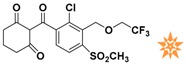	2.216	372.82	6	0	5	102.95	0.01
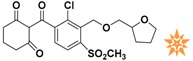	2.827	369.39	7	2	4	137.48	0.01
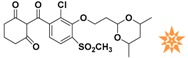	1.883	328.77	5	1	3	93.72	0.01
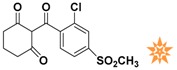	2.336	442.91	7	1	7	115.35	0.01
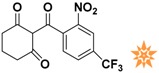	1.965	458.91	8	1	6	124.57	0.04
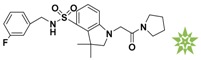	3.086	445.55	4	1	6	78.09	-
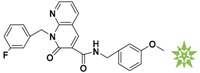	3.371	417.43	4	1	6	71.53	-
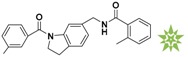	4.659	384.47	2	1	4	49.41	-
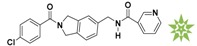	3.629	391.85	3	1	4	62.3	-

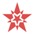
 represents the ligand in the crystallographic complex, 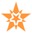 represents the training set compounds in the HipHop model, and 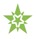 represents the hit compounds identified by virtual screening.

**Table 3:** Chemical feature compositions for the ten hypotheses generated from six known HPPD inhibitors.

Hypothesis	Features	Rank
HipHop-Hypo1	RHAAAA	81.854
HipHop-Hypo2	RHAAAA	81.854
HipHop-Hypo3	RHAAAA	81.788
HipHop-Hypo4	RAAAA	72.750
HipHop-Hypo5	RAAAA	72.347
HipHop-Hypo6	RAAAA	72.293
HipHop-Hypo7	RAAAA	72.234
HipHop-Hypo8	RAAAA	72.188
HipHop-Hypo9	RAAAA	72.188
HipHop-Hypo10	RAAAA	72.167

R (ring aromatic), H (hydrophobic features), A (Acceptor).
